# Case Report: A typical triad of Danon disease caused by a LAMP2 splice-donor variant with multilevel functional validation

**DOI:** 10.3389/fcvm.2026.1695991

**Published:** 2026-05-07

**Authors:** Yang Hu, Shijie Liu, Qian Liu, Bosu Liu, Jinting Zhang, Huipeng Su, Li Yang, Lulu Gan, Yangfan Guo, Yan He

**Affiliations:** 1 Heart Failure Center, Department of General Practice, Yan 'an Hospital Affiliated to Kunming Medical University, Kunming, China; 2 Central Laboratory, Yan 'an Hospital Affiliated to Kunming Medical University, Kunming, China; 3 Organ Procurement Organization, Yan 'an Hospital Affiliated to Kunming Medical University, Kunming, China; 4 Precision Medicine Center, Yan 'an Hospital Affiliated to Kunming Medical University, Kunming, China; 5 Key Laboratory of Tumor Immunological Prevention and Treatment of Yunnan Province, Kunming, China; 6 Key Laboratory of Cardiovascular Disease of Yunnan Province, Kunming, China; 7 Yunnan Provincial Clinical Research Center for Cardiovascular Disease, Kunming, China

**Keywords:** cardiomyopathy, Danon disease, intellectual disability, LAMP2, skeletal myopathy, splice-site variant

## Abstract

Danon disease is a rare X-linked dominant disorder caused by pathogenic variants in *LAMP2*, typically presenting with cardiomyopathy, skeletal myopathy, and intellectual disability, and showing a severe course in males. In this study, we report the case of a 29-year-old Han Chinese male with the classic triad, plus macular degeneration and a complex neuromuscular phenotype including axonal–demyelinating sensorimotor polyneuropathy. Whole-exome sequencing identified a hemizygous splice-donor variant in *LAMP2* (NM_002294.2:c.928 + 1G > A). A functional analysis in peripheral blood with matched controls experimentally confirmed markedly reduced *LAMP2* mRNA levels and decreased LAMP2 protein expression, supporting the variant as a loss-of-function allele. The patient rapidly progressed to end-stage heart failure and died 18 months after diagnosis, highlighting the severe multisystem impact of this variant. This case expands the functional evidence for pathogenic *LAMP2* splice-site variants and suggests peripheral nervous system involvement in severe multisystem Danon disease.

## 1 Introduction

Danon disease is a rare, X-linked dominant genetic disorder caused by pathogenic variants in the *lysosomal-associated membrane protein 2 (LAMP2)* gene (1). Deficiency of the LAMP2 protein, which is essential for autophagosome–lysosome fusion, disrupts autophagy, leading to the accumulation of glycogen and autophagic vacuoles in affected tissues, particularly cardiac and skeletal muscle (2). Clinically, it is characterized by a classic triad of cardiomyopathy, skeletal myopathy, and variable intellectual disability, with male patients typically exhibiting a more severe and rapidly progressive phenotype (3).

In this study, we report the case of a 29-year-old male with classical clinical presentation of Danon disease, in whom whole-exome sequencing identified a pathogenic variant in LAMP2 (NM_002294.2:c.928 + 1G > A). This case provides a significant contribution by extending beyond the typical triad to provide a detailed characterization of a complex neuromuscular phenotype, including a concurrent axonal–demyelinating sensorimotor polyneuropathy. Furthermore, we perform a robust, control-based functional validation using RNA sequencing, quantitative PCR, and Western blotting, which demonstrates that this variant leads to a profound loss of *LAMP2* transcript via nonsense-mediated decay (NMD) and a complete absence of the protein. Our findings therefore not only expand the pathogenic mutational spectrum of *LAMP2* but also refine the understanding of its peripheral nervous system involvement, linking this null-type variant to a severe and fatal multisystem clinical course of Danon disease.

## 2 Case presentation

**Figure 1:**
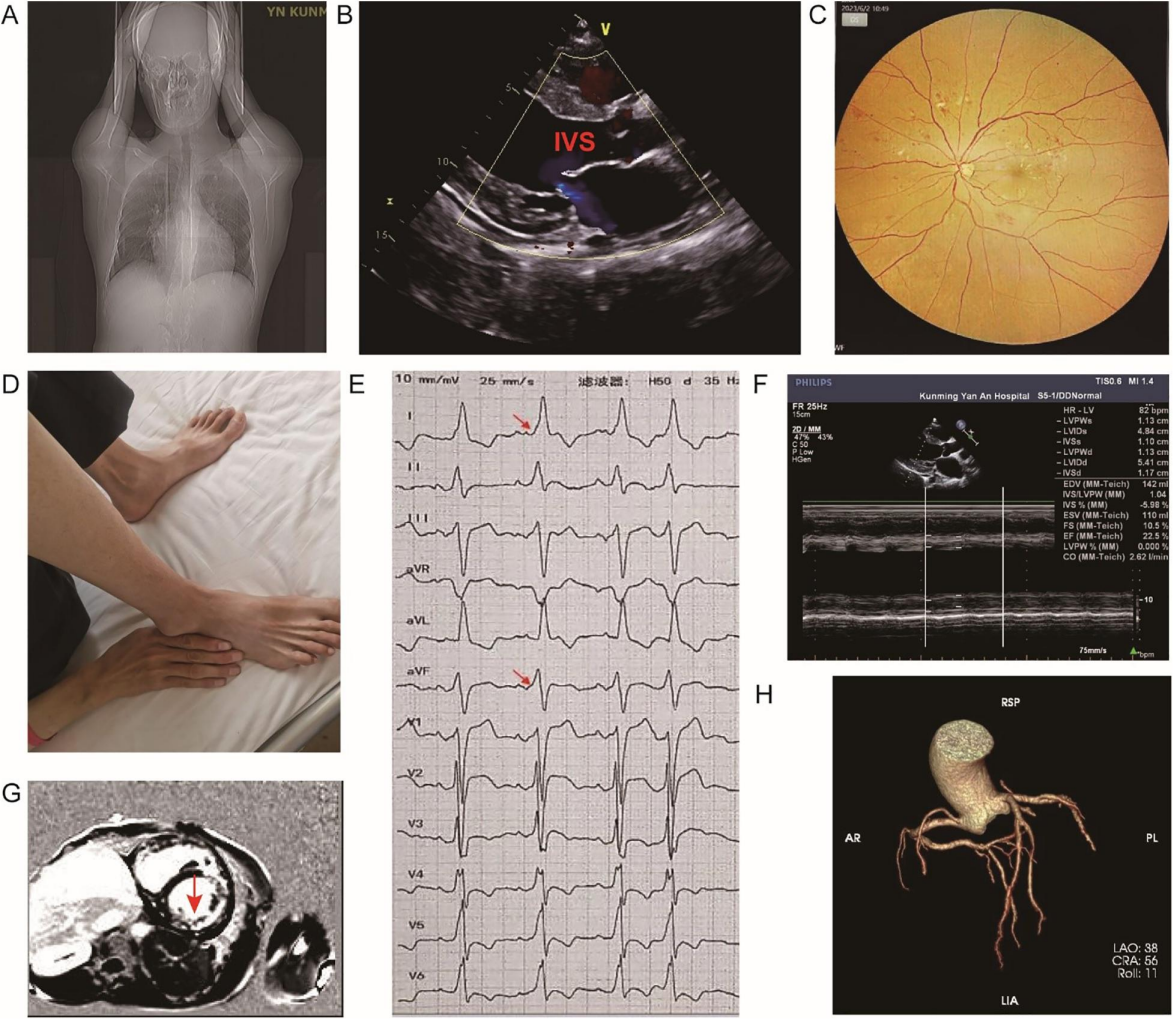
Major clinical findings during the examination of the patient. **(A)** A CT scout view demonstrating significant cardiomegaly. **(B)** Echocardiography showing a markedly thickened interventricular septum(IVS). **(C)** Fundus photography revealing macular pathology with pigmentary changes. **(D)** A clinical photograph showing skeletal muscle wasting and weakness. **(E)** An ECG showing ventricular preexcitation (Wolff–Parkinson–White pattern) with a short PR interval and delta waves (red arrow). **(F)** Echocardiography demonstrating severely impaired cardiac function with a left ventricular ejection fraction (LVEF) of 22.5%. **(G)** CMR with LGE showing diffuse myocardial fibrosis in the LV free wall, with characteristic sparing of the interventricular septum (red arrow), and the percentage of fibrosis was quantified at 21%. **(H)** A 3D volume–rendered reconstruction from CCTA confirming normal coronary arteries.

A 29-year-old, unmarried Han Chinese man residing in Yunnan, presented with a 5-year history of progressive exertional dyspnea and fatigue, which ultimately culminated in an inability to perform daily activities. He had a history of mild intellectual disability (Wechsler Adult Intelligence Scale score: 54). A physical examination revealed slurred speech and an enlarged cardiac border on percussion (Figures 1A, B), and a fundoscopic examination showed evidence of macular degeneration (Figure 1C). A neurological examination with Lovett's 6-level muscle strength assessment (4) revealed a symmetrical limb-girdle pattern of weakness, with muscle strength graded 3/5 proximally and 4/5 distally. In contrast to this distribution of weakness, visible muscle atrophy was more prominent distally (Figure 1D). Further assessment identified mild dysphagia (Grade 2, Watada water drinking test) and a mild restrictive ventilatory defect on pulmonary function testing (FVC, 68.3% of predicted; TLC, 74.6% of predicted), indicative of early bulbar and respiratory muscle involvement.

Electromyography and nerve conduction studies subsequently confirmed axonal–demyelinating sensorimotor polyneuropathy. The electrophysiological profile demonstrated diffuse involvement of motor nerves (axonal and demyelinating features) and sensory nerves (predominantly demyelinating) in both upper and lower limbs. Taken together with EMG evidence of neurogenic changes, these results support a complex neuromuscular process with proximal and distal involvement and possible root-level pathology.

Laboratory investigations revealed a profound biochemical derangement consistent with multiorgan pathology, characterized by substantial elevations in markers of cardiac stress and injury (N-terminal pro-B-type natriuretic peptide, 2769 pg/mL; high-sensitivity cardiac troponin I, 0.075 µg/L), extensive myolysis (creatine kinase, 899 U/L; myoglobin, 480.3 µg/L; lactate dehydrogenase, 997 U/L), and concurrent hepatic cytolysis (alanine aminotransferase, 246 U/L; aspartate aminotransferase, 190 U/L).

The admission electrocardiogram (ECG) revealed a sinus rhythm at 82 bpm with frequent supraventricular premature beats, evidenced by a short PR interval and a delta wave (Figure 1E). Echocardiography further demonstrated severe biventricular enlargement, concentric left ventricular hypertrophy, and a severely reduced ejection fraction (22.5%) (Figure 1F). Cardiac magnetic resonance (CMR) revealed a distinctive pattern of diffuse, subendocardial late gadolinium enhancement (LGE) throughout the left ventricular free wall, with characteristic sparing of the interventricular septum (Figure 1G), and the percentage of fibrosis was quantified at 21%. 3D volume-rendered reconstructions (Figure 1H) confirmed patent coronary arteries, ruling out an ischemic etiology.

**Figure 2:**
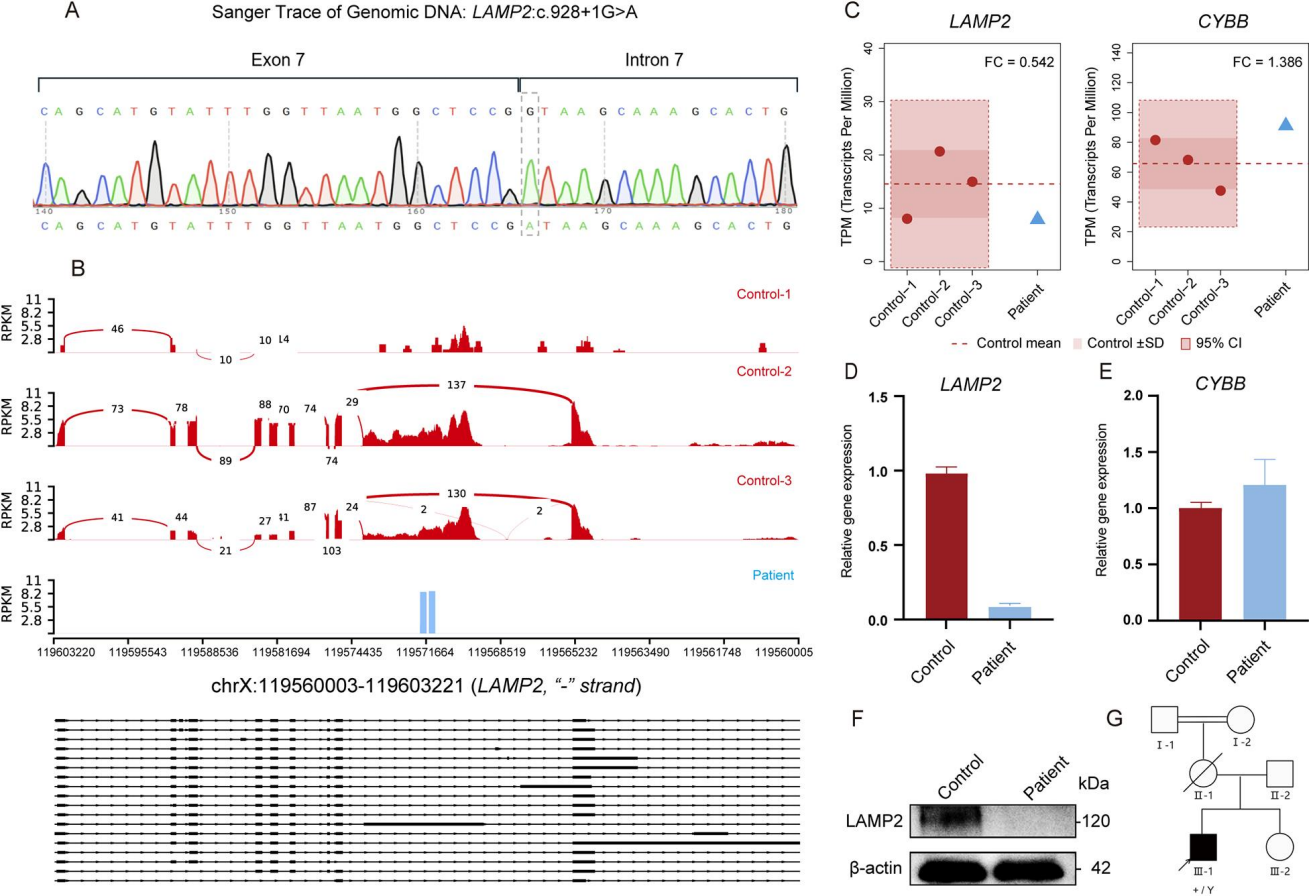
Genetic and functional validation of the *LAMP2* splice-site variant. **(A)** Sanger sequencing confirms the hemizygous G > A substitution at the canonical +1 position of the exon 7 splice-donor site. **(B)** An RNA-seq was performed on total RNA extracted from peripheral blood samples. Libraries were prepared using standard protocols and sequenced on an Illumina platform. Clean reads were obtained after quality control and aligned to the reference genome using HISAT2, followed by gene expression quantification with featureCounts. Expression levels were normalized as TPM. RNA-seq Sashimi plots reveal markedly reduced *LAMP2* read coverage and splicing junctions in the patient compared with three healthy male controls. **(C)** A transcript quantification of *LAMP2* and *CYBB* using TPM (Transcripts Per Million), normalized for sequencing depth and gene length. *LAMP2* expression in the patient falls within the lower range of controls but is below the mean level, while *CYBB* expression remains comparable. **(D)** An RT-qPCR was performed on cDNA reverse-transcribed from total RNA extracted from peripheral blood samples. Reactions were carried out using SYBR Green master mix with gene-specific primers for LAMP2 (located within exon 8), and expression levels were normalized to the reference gene *CYBB*. Relative mRNA expression was calculated using the 2^−ΔΔCt^ method. All measurements were performed using technical replicates. An RT-qPCR analysis showing reduced *LAMP2* mRNA levels in the patient relative to controls, **(E)** while the expression of the *CYBB* control remains unchanged. **(F)** A Western blot analysis was performed on total protein extracted from peripheral blood cells. The proteins were separated by SDS–PAGE, transferred onto PVDF membranes, and incubated using an anti-LAMP2 primary antibody (Proteintech, Cat# 27823-1-AP) followed by appropriate secondary antibodies. Protein signals were detected using an enhanced chemiluminescence system. No detectable LAMP2 protein is observed in the patient. **(G)** The family pedigree identifying the proband (III-1, arrow) with the hemizygous variant.

Whole-exome sequencing was performed to elucidate the genetic etiology of this multisystem phenotype. The sequencing result identified a hemizygous variant in the *LAMP2* gene (NM_002294.2:c.928 + 1G > A), which was subsequently confirmed by Sanger sequencing (Figure 2A). This variant affects the highly conserved canonical +1 donor splice site of intron 7 and is predicted by multiple splicing tools to abolish normal splicing, likely resulting in exon 7 skipping (PVS1). It is absent from the gnomAD v2.1.1 database (PM2_supporting) and was previously reported as DM in HGMD (5). In addition, it was identified in a patient with dilated cardiomyopathy, and a different nucleotide substitution at the same splice site (NM_002294.3:c.928 + 1G > T) was also classified as pathogenic (PS1_supporting) (6). The patient in our study exhibited the classic triad of Danon disease (PP4). According to ACMG guidelines, this variant is classified as pathogenic (PVS1 + PS1_supporting + PM2_supporting + PP4).

To evaluate the functional consequence of the LAMP2:c.928 + 1G > A variant, we analyzed peripheral blood from the patient and three age- and sex-matched healthy controls. RNA sequencing was performed on total RNA extracted from peripheral blood samples. Gene expression levels were normalized as Transcripts Per Million, accounting for sequencing depth and gene length. An RNA-seq analysis revealed a marked reduction in *LAMP2* read coverage and a loss of canonical splice junction support in the patient compared with controls (Figure 2B). Notably, low levels of residual reads were still detectable, suggesting that the mutant transcript is not completely absent but substantially reduced, consistent with partial degradation mechanisms such as NMD. Transcript quantification showed that *LAMP2* expression in the patient overlapped with the lower range of controls but was below the mean level of the control group, indicating an overall reduced trend (Figure 2C). Crucially, the X-linked reference gene *CYBB* exhibited robust expression comparable to controls in both RNA-seq (Figure 2C) and RT-qPCR assays (Figure 2E). An RT-qPCR analysis was performed using cDNA reverse-transcribed from total RNA extracted from peripheral blood samples. Gene-specific primers for LAMP2 (forward: CCCCTGGGAAGTTCTTATATGTG; reverse: GTCACATTGAAAGGCTGAACC), located within exon 8, were used for amplification. Expression levels were normalized to the reference gene CYBB. All reactions were performed with technical replicates. RT-qPCR showed reduced *LAMP2* mRNA levels in the patient compared with controls (Figure 2D). Notably, using primers spanning the Exon 8 junction, we found that amplification in the patient was markedly reduced compared with controls, which was consistent with reduced levels of the canonical transcript. For protein analysis, total protein was extracted from peripheral blood cells using lysis buffer. Protein concentrations were determined to ensure equal loading. Samples were separated by SDS–PAGE and transferred onto PVDF membranes. Membranes were incubated using an anti-LAMP2 primary antibody (Proteintech, Cat# 27823-1-AP), followed by appropriate secondary antibodies. Protein signals were detected using a chemiluminescent substrate. No detectable LAMP2 protein was observed in the patient (Figure 2F). Collectively, these results indicate that the splice-site variant likely triggers NMD, resulting in a null allele and total loss of LAMP2 expression.

The patient's clinical course was refractory to guideline-directed medical therapy, and the patient achieved only transient symptomatic relief, while exhibiting persistent elevations in muscle and liver enzymes. This aggressive clinical trajectory was consistent with the severe familial disease course suggested by the medical history of his mother (II-1, Figure 2G), the presumed carrier. According to the proband, his mother developed progressive fatigue, chest tightness, and dyspnea around 40 years of age and died in 2009 at the age of 46 from heart failure of unknown etiology. Her carrier status was inferred based on the strong phenotypic overlap and X-linked inheritance, as genetic confirmation was not possible posthumously. The proband ultimately progressed to end-stage heart failure and, after declining a recommended heart transplant, succumbed to the disease 18 months after diagnosis. Written informed consent for publication was obtained from the proband's father.

## 3 Discussion

Herein, we presented a case of a patient with Danon disease associated with a hemizygous pathogenic variant (c.928 + 1G > A) in the *LAMP2* gene, which offers a compelling explanation for the patient's severe and rapidly progressive clinical course. The *LAMP2* gene encodes three main protein isoforms (LAMP2A, LAMP2B, and LAMP2C) through the alternative splicing of its terminal exon. The LAMP2B isoform, which is predominantly expressed in the cardiac and skeletal muscles, is indispensable for the fusion of autophagosomes with lysosomes—a critical step in cellular autophagy (7). Given its location upstream of this alternatively spliced region, the identified variant is predicted to cause a global loss of LAMP2 function, particularly the abolition of LAMP2B-mediated autophagy in cardiomyocytes, therefore directly accounting for the patient's profound hypertrophic cardiomyopathy and early mortality, aligning with the most severe outcomes reported for Danon disease (8).

The *LAMP2* c.928 + 1G > A variant abolishes the canonical splice-donor site of intron 7. This disruption is predicted to cause aberrant splicing (e.g., exon 7 skipping), which would introduce a frameshift and a subsequent premature termination codon. Consequently, the resulting mutant transcript is then targeted for degradation by the NMD pathway, resulting in significantly diminished but detectable transcript levels. Crucially, this case demonstrates the necessity of multilevel functional validation. Although the variant has been previously catalogued in HGMD without comprehensive functional characterization (5), our integrative analysis combining RNA sequencing, qPCR, and Western blotting offers compelling evidence elucidating its pathogenic mechanism. RNA-seq with healthy controls showed reduced *LAMP2* read coverage and loss of canonical splice junction support, while RT-qPCR confirmed transcript depletion consistent with NMD. Isoform-aware quantification further demonstrated concordant reduction across the major isoforms (LAMP2A/B/C). A Western blot analysis confirmed the complete absence of LAMP2 protein, supporting a functional null allele. Collectively, these findings provide a mechanistic explanation for the severe, rapidly progressive multisystem phenotype in this patient.

**Figure 3:**
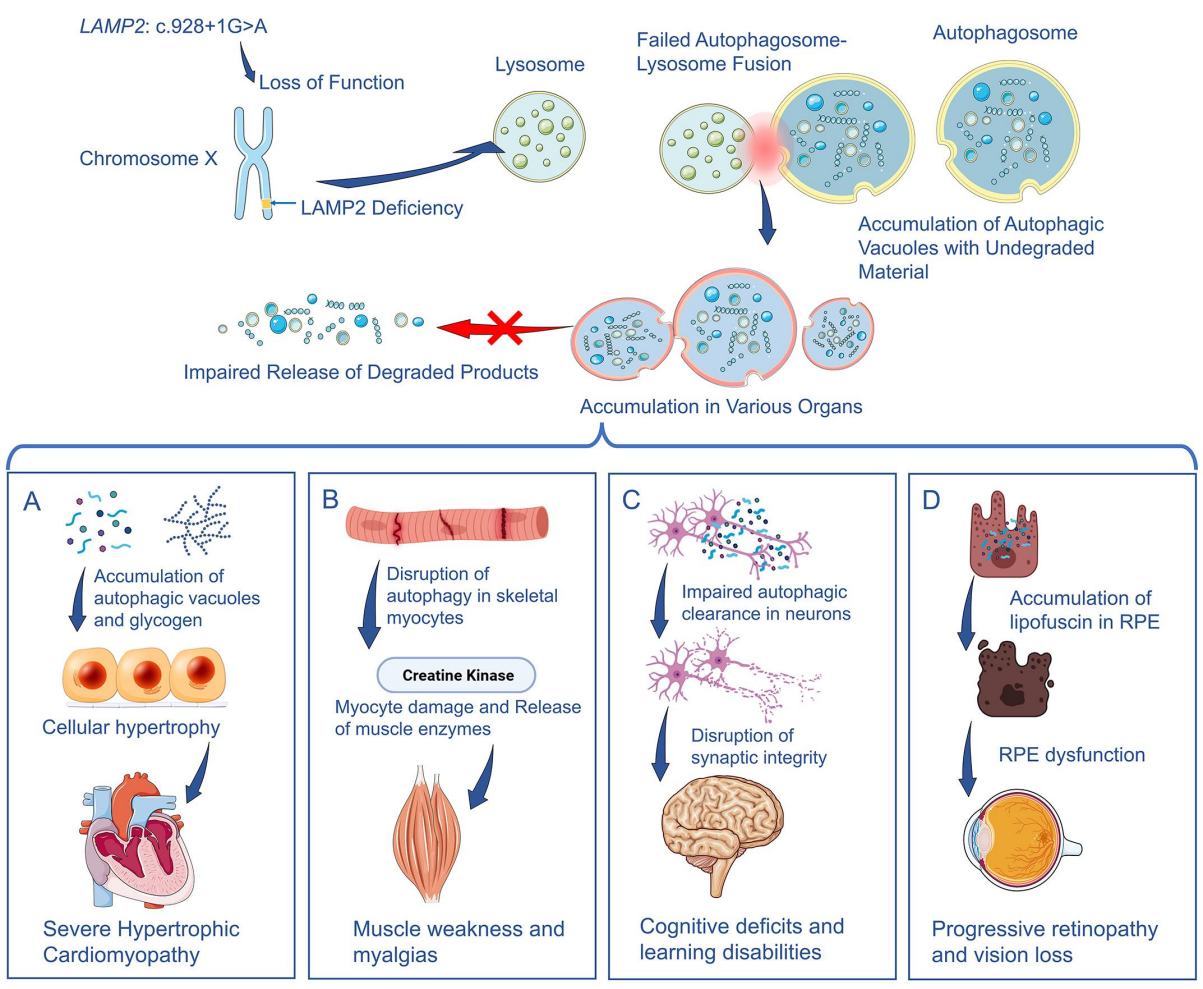
Pathophysiological mechanisms of Danon disease. A loss-of-function variant (c.928 + 1G > A) in *LAMP2* leads to impaired autophagosome–lysosome fusion and accumulation of autophagic vacuoles. This defect drives the characteristic multiorgan pathology: **(A)** Accumulation of autophagic vacuoles and glycogen within cardiomyocytes leading to severe cardiomyopathy. **(B)** The disruption of autophagy in myocytes leading to myocyte damage and resulting in muscle weakness. **(C)** Impaired autophagic clearance in neurons contributing to cognitive deficits. **(D)** Retinal pigment epithelium (RPE) damage leading to the accumulation of lipofuscin and causing progressive retinopathy.

The global loss of LAMP2 function predicted from the c.928 + 1G > A variant provides a unified molecular basis for the classic triad phenotype of the patient. Cardiac involvement was severe and rapidly progressive, with marked hypertrophic cardiomyopathy, end-stage heart failure, and early death. This is consistent with impaired LAMP2B-dependent autophagosome–lysosome fusion, which promotes vacuole and glycogen accumulation in cardiomyocytes and leads to hypertrophy and replacement fibrosis (Figure 3A) (9). Accordingly, CMR in our patient showed diffuse subendocardial LGE with septal sparing and a high fibrosis burden (Figure 1G). The patient's muscle phenotype—predominant limb-girdle weakness with marked distal wasting and persistent hyperCKemia—fits the typical vacuolar myopathy of Danon disease, in which impaired autophagosome–lysosome fusion causes vacuole accumulation and progressive myofiber damage (Figure 3B) (10). Moreover, the patient's mild intellectual disability highlights the critical role of autophagy in the central nervous system. Impaired autophagic clearance in neurons disrupts synaptic integrity and long-term neuronal health, contributing to the observed cognitive deficits (Figure 3C) (11). This same principle of autophagic disruption extends to the patient's retinopathy. The retinal pigment epithelium (RPE) relies heavily on efficient autophagy to degrade photoreceptor outer segments, and failure of this process leads to the accumulation of lipofuscin and other cellular debris, causing RPE dysfunction and progressive vision loss (Figure 3D) (12).

The patient's fatal outcome at the age of 29 highlights the poor prognosis typically associated with male Danon disease; males typically progress to end-stage heart failure or require cardiac transplantation by their third decade of life (13). This stark sexual dimorphism is a direct consequence of its X-linked inheritance: males, being hemizygous, lack a compensatory *LAMP2* allele, whereas heterozygous females exhibit a milder, more variable phenotype because of random X-chromosome inactivation (14). This clinical divergence creates a significant ascertainment bias, leading to a paradoxical male predominance in clinical registries that contradicts the expected 2:1 female-to-male ratio (15). This discrepancy is largely attributed to a significant ascertainment bias: the severe, early-onset phenotype in males leads to more frequent and accurate diagnoses, while the often-subtle presentation in females leads to under- or misdiagnosis as other forms of cardiomyopathy.

The absence of any approved disease-modifying therapy for Danon disease restricts clinical management to supportive care, primarily aimed at mitigating heart failure symptoms and preventing sudden cardiac death with device therapy (16). For end-stage disease, cardiac transplantation is the sole definitive option, offering an 80% 5-year survival rate but remaining accessible to only approximately 17.6% of patients (17). Given its monogenic etiology, gene replacement therapy targeting the *LAMP2* family gene represents the most promising investigational strategy. The leading candidate, RP-A501, is an AAV9-vectored therapy delivering a functional *LAMP2B* transgene; preliminary data from its Phase I/II trial (NCT03882437) (18, 19) are encouraging, showing reductions in cardiac stress biomarkers and improvements in myocardial function. In parallel, pharmacological strategies, such as mTOR inhibitors, and the use of iPSC-derived disease models for drug screening continue to expand the therapeutic pipeline (20, 21). Ultimately, the advancement of precision genome editing tools holds the greatest potential for a permanent cure by directly correcting the causative *LAMP2* mutation.

## 4 Conclusion

This report identifies and functionally validates a pathogenic *LAMP2* splice-site variant (c.928 + 1G > A) as the cause of a fatal case of Danon disease. Using a rigorous, control-based experimental framework, we demonstrate that this variant disrupts canonical splicing and results in a marked depletion of *LAMP2* transcript and reduction of LAMP2 protein expression, consistent with a null allele. This case expands the known pathogenic mutational spectrum of Danon disease and highlights the value of functional transcriptomic analyses in strengthening the evidence supporting variant pathogenicity.

## Data Availability

The datasets presented in this study can be found in online repositories. The names of the repository/repositories and accession number(s) can be found in the article/Supplementary Material.
